# Best Practice Guidance for Male Individuals Using Anabolic Androgenic Steroids in Recreational Sports Within Primary Care: Protocol for a Modified Delphi Consensus Study

**DOI:** 10.2196/65233

**Published:** 2025-08-18

**Authors:** Raphael Magnolini, Oliver Senn, Stefan Neuner-Jehle, Philip Bruggmann

**Affiliations:** 1 Institute of Primary Care University of Zurich and University Hospital Zurich Zurich Switzerland; 2 Arud Centre for Addiction Medicine Zurich Switzerland

**Keywords:** anabolic androgenic steroids, doping, image- and performance-enhancing drugs, primary care, study protocol, protocol, male, user, steroids, recreational sports, Delphi consensus, androgenic steroid, questionnaire technique

## Abstract

**Background:**

The nonmedical use of anabolic androgenic steroids (AASs) has become a considerable substance use concern worldwide, and it is a growing public health risk, particularly among male individuals in recreational sports. Individuals who use these substances for recreational purposes may experience various medical problems. However, there is a lack of high-quality guidance and consensus among medical experts regarding the adequate provision of medical care for this population.

**Objective:**

This study aims to develop best practice guidance for health care professionals providing medical care to individuals engaging in the nonmedical use of AASs by achieving consensus on relevant and feasible measures for the Swiss primary care context.

**Methods:**

The Delphi consensus method will be applied in this study to develop best practice guidance, following the criteria for conducting and reporting Delphi studies (DELPHISTAR). The development of evidence-based, relevant, and feasible measures for primary care practice follows a mixed methods approach to identify the problem area by conducting 4 substudies: a scoping literature review, incorporation of medical experience, peer involvement, and the selection of a heterogeneous research steering group of medical experts based on individual and group discussions. This Delphi consensus study consists of 3 rounds of anonymous web-based surveys with medical experts in primary care (N=25 Delphi panelists). Recruitment will occur through a network of primary care experts at the University of Zurich. Participants will rate each measure for relevance and feasibility using a 5-point Likert scale and provide comments and recommendations for additional measures. Each measure will require more than 75% agreement in both relevance and feasibility to achieve consensus. Descriptive statistics will be used. Aggregated ratings will be statistically analyzed for response rates, level of agreement, medians, and IQRs. Qualitative responses will be analyzed thematically and evaluated by the steering group for inclusion in subsequent rounds. The final draft of the resulting best practice guidance will be reviewed by an external board of medical associations, and approval will be sought before publication and dissemination.

**Results:**

The resulting best current clinical guidance is expected to be published in the summer of 2026. Ethics approval was received from the local ethics committee for this study.

**Conclusions:**

With the development of high-quality best practice guidance for health care professionals in primary care, this Delphi study will help close the existing treatment gap among people using AASs. As a strength, primary care offers low-threshold access to health care, chronic care management, and coordination within the health care sector for patients experiencing multiple AAS-related health complications. The Delphi technique is an appropriate study design to develop consensus among a group of medical experts, with the strength of maintaining anonymity throughout the survey process.

**International Registered Report Identifier (IRRID):**

PRR1-10.2196/65233

## Introduction

### Background

The nonmedical use of anabolic androgenic steroids (AASs) and other image- and performance-enhancing drugs (IPEDs) for optimizing personal esthetic goals and improving sports performance represents one of the newest major global substance use concerns [1-4]. IPEDs comprise different groups of substances, with AASs being the most commonly used [2,3,5-9]. According to a systematic review and meta-analysis, the global lifetime prevalence of AAS use is estimated to be 3.3% in the general population, 6.4% among male individuals, and 18.4% among recreational sportspeople [10]. Considering that the popularity and prevalence of IPED use may have increased in recent years, the problematic use of these substances should be considered a serious and growing risk for public health [4,5]. In Switzerland, the use of anabolic agents appears to be widespread, with an estimated 200,000 to 300,000 users [11,12]. IPEDs can easily be acquired from unregulated drug markets [8,13].

Acute and long-term complications of AAS use can be complex, potentially severe, and life-threatening, affecting all levels of health (ie, physical, mental, and social health) [3,5,14-16]. Importantly, the mortality from natural and unnatural causes among people who use AASs appears to be higher than that in the general population [17-20]. The great extent of harms associated with AAS consumption emphasizes the urgent need for comprehensive and integrated treatment and harm-reduction strategies.

An extensive treatment gap may exist for AAS users. Most people using AASs report experiencing side effects and complications from the use of these substances, but only a minority group of people appear to attend medical care, even though these services are commonly desired by AAS users [3,21-24]. Patients who access medical care often do not disclose the use of these substances due to fear of stigma and lack of trust in health care professionals, leading to inadequate care and decision-making by health care professionals [2,3,22]. Most often, people who use AASs report self-medicating for medical problems, seeking information and support from nonmedical sources (ie, experienced users and peers or online forums) [3,22,25,26].

Further barriers include existing legal restrictions for the provision of medical care for users of AASs worldwide [27,28]. IPEDs, in the context of sports or “doping,” are banned by the World Anti-Doping Agency and prohibited by regulatory agencies [8,13]. Doping policies in Switzerland can criminalize physicians who provide medical care to these patients under certain circumstances [11,29]. Health care providers face legal uncertainty when providing care for patients using AASs, and legal aspects can hinder open discussion and engagement with health care providers from a patient perspective [30,31]. Knowledge of antidoping regulations is crucial to help physicians avoid legal problems and address their concerns.

### Objectives

In recent years, a few clinics in Europe have established novel care models that aim to provide medical care and harm-reduction services for this population, and important clinical and health research data have emerged [7,32-37]. Despite these advances, many knowledge gaps remain regarding the optimal provision of care, particularly within a primary care context. Although some expert opinions are available in the published literature, current practice guidance is often not comprehensive, focuses on one disease area, demonstrates a lack of consensus among health care experts, and challenges clinical practice [2,7,32-42]. This study aims to develop best practice guidance for health care professionals providing medical care to individuals engaging in the nonmedical use of AASs by achieving consensus on relevant and feasible measures for a Swiss primary care context.

## Methods

### Justification for Study Design

For the development of best practice guidance, the Delphi method will be used. The Delphi method is a questionnaire technique that uses multiple iterations designed to build a justified consensus concerning a specific topic among an expert panel [43,44]. It has played a pivotal role over the past few decades in addressing research gaps within health science. The development of best practice guidance is achieved using collective intelligence in areas where research and high-quality data are limited. The strengths and suitability of the Delphi technique for this study include participant anonymity and flexible design compared to other consensus models (eg, focus groups) [44].

This Delphi study will focus solely on AAS use based on the epidemiology of use and the development of androgen dependence syndrome [3,45,46]. Notably, the use of AASs compared to other anabolic agents and IPEDs is most prevalent [3,6]. Substances other than AASs are beyond the scope of this research.

### Methodology and Reporting Guidelines

The critical appraisal of a Delphi study in health care follows the methodological process suggested by Nasa et al [47]. The conduct of this study follows the DELPHISTAR (Delphi studies in social and health sciences–recommendations for an interdisciplinary standardized reporting) criteria for conducting and reporting Delphi studies [48].

### Scope and Context for Use

The development of best practice guidance is aimed at general practitioners in primary care settings in Switzerland who provide medical care for male users of AASs in recreational sport (ie, noncompeting recreational sportspeople, weightlifters, or bodybuilders). The provision of medical care for competitive athletes (ie, those who belong to a control pool within the framework of the World Anti-Doping Code) is outside the legal scope of this project.

### Study Setting

This study will be conducted collaboratively by the Arud Centre for Addiction Medicine in Zurich, the Institute of Primary Care at the University of Zurich, and the University Hospital of Zurich. The Arud Centre established integrated primary care and treatment services for users of AASs in Zurich in June 2023 [29]. In Switzerland, the Arud Centre provides integrated medical care services for users of AASs, including general internal medicine, psychiatry, and addiction medicine. The Institute for Primary Care in Zurich provides expert opinion on health service research, focusing on integrated care in the primary care setting.

### Project Governance

A project governance group has been established with the responsibility of overseeing the conduct of the project. The group consists of representatives from the Arud Centre (RM and PB) and the Institute of Primary Care (OS and SN-J).

### Study Design

**Figure 1:**
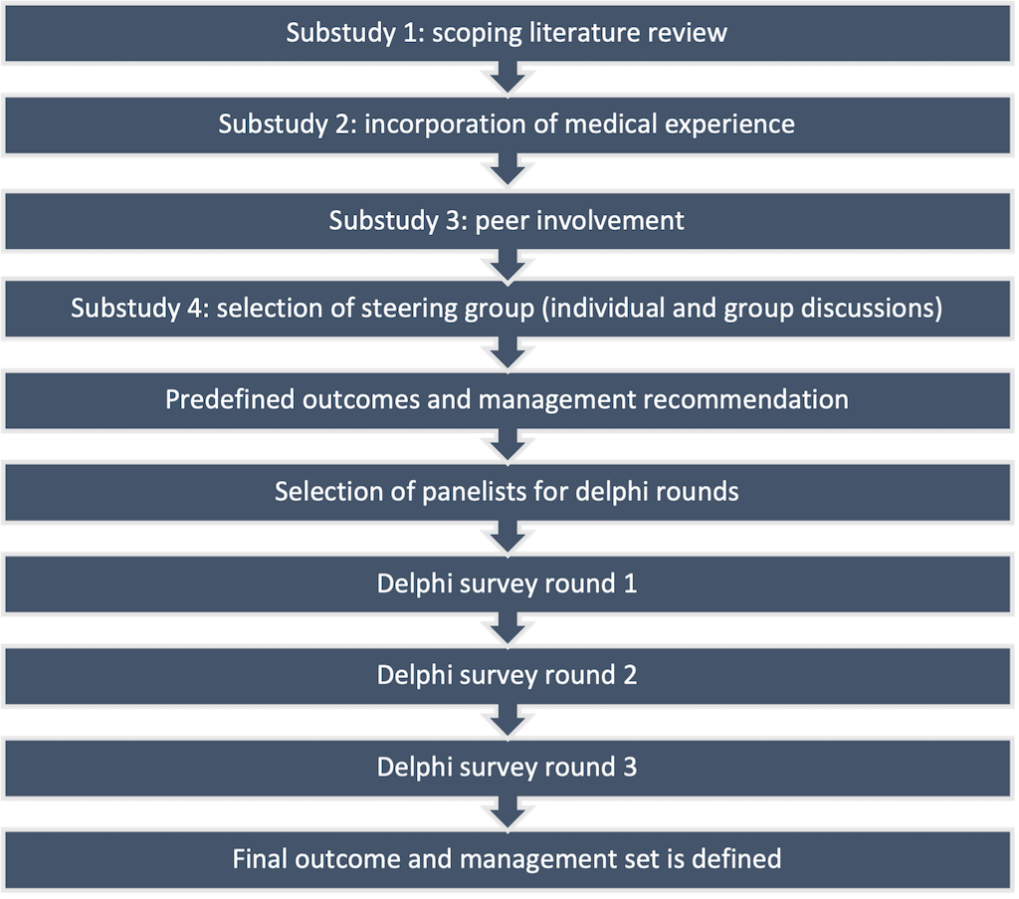
Delphi study workflow.

The Delphi consensus study is conducted sequentially in a four-step methodological process, including (1) identification of the problem area (mixed methods approach), (2) selection of the panel (medical experts), (3) iterative Delphi rounds, and (4) closing criteria (consensus and primary outcome). The complete workflow for this study is presented in Figure 1.

### Systematic Identification of Problem Area

The identification of problem area will be achieved by conducting four substudies, including (1) a scoping literature review, (2) incorporation of medical experience, (3) peer involvement, and (4) selection of a research steering group with individual and group discussions.

#### Substudy 1: Scoping Literature Review of Peer-Reviewed Medical Literature

A scoping literature review will be conducted to systematically identify, evaluate, and synthesize existing research related to the following:

1. How to screen for and diagnose AAS use
2. Mapping side effects and complications from AAS use
3. Mapping management recommendations for side effects of and complications from AAS use

This scoping review will follow the PRISMA-ScR (Preferred Reporting Items for Systematic Reviews and Meta-Analyses extension for Scoping Reviews) guidelines (Multimedia Appendix 1 [49]). The eligibility criteria are established to ensure relevance and quality. The inclusion criteria comprise peer-reviewed articles published between 2005 and 2025 in English and that were related to the aforementioned topics. The exclusion criteria comprise non–peer-reviewed articles, non-English publications, and articles unrelated to the study topics.

A comprehensive search strategy will be undertaken in electronic databases (ie, PubMed, MEDLINE, and Embase). Additional sources, such as reference lists of identified articles, will also be searched to supplement the database results. Furthermore, existing guidelines from medical societies will be assessed for management recommendations (ie, testosterone replacement therapy). The search strategy will involve structured keywords and Boolean operators and can be found in Multimedia Appendix 2.

Two independent and experienced reviewers will screen titles and abstracts for relevance based on the eligibility criteria. Full-text articles of potentially relevant articles will be obtained and reviewed independently by the same reviewers. The proposed explicit consensus criteria in the final paper will reference the available evidence where possible. This will be achieved by the research steering group undertaking a detailed literature review following the recommendations and processes set out by the Grading of Recommendations Assessment, Development, and Evaluation guidelines [50].

A standardized data extraction form will be used to chart relevant data from the included studies. The form will capture study characteristics (ie, author, year, and country), study design and methodology, and principal findings related to the research question. The extracted data will be analyzed using a thematic synthesis approach. The findings will be organized into themes and subthemes to provide an overview of the current state of research on the topics. Quality assurance of individual studies will not be assessed as per the typical scoping review methodology. However, the review process will be documented in detail to ensure transparency. The results will be presented descriptively, using tables and figures to illustrate key findings and thematic categorizations.

#### Substudy 2: Medical Experience

In Switzerland, a primary health care practice for users of AASs was established in 2023 at the Arud Centre for Addiction Medicine in Zurich, in collaboration with the Institute for Primary Care at the University of Zurich [29]. Experiences and data derived from this health care service will be used to support the literature review and fill existing knowledge gaps.

#### Substudy 3: Peer Involvement

Peer support services within addiction medicine, which incorporate peers with lived experiences, have been used in various clinical settings to assist individuals with substance use disorders [51]. Peers among people using AASs currently play a crucial role in providing information about use and offering harm-reduction advice for this population [2,3,22]. There is a need to incorporate lived experiences from users and engage peers in developing comprehensive harm-reduction strategies. Outcomes and management recommendations established through previous steps will be reviewed by a selected group of peers (n=5) before being finalized for use in the Delphi rounds. The selection of peers will occur from the established patient cohort at the Arud Centre [29].

#### Substudy 4: Selection of the Research Steering Group With Individual and Group Discussions

A research steering group will be selected by the study team to conduct this research. They will be responsible for preparing and circulating the content of the Delphi rounds. The research steering group will comprise heterogeneous medical experts and health care professionals with various backgrounds. The selection of experts is based on outcomes and disease areas established during the previous steps (eg, cardiology, psychiatry, and endocrinology). Selection criteria for steering group members include expertise in managing side effects and complications from the use of anabolic steroids, as well as holding a Foederatio Medicorum Helveticorum (Swiss Medical Association) specialization or title in their respective field or certification or license in their respective fields, where applicable (eg, addiction medicine, sports medicine, and andrology). The research steering group will not participate in the surveys; they will supervise and monitor the process and provide expert guidance. Outcomes and management recommendations established through previous steps will be reviewed by the expert steering group before being finalized for use in the Delphi rounds. Disagreements will be resolved by consensus among the steering group members through individual and group discussions for the final draft to be used in the Delphi rounds.

### Selection and Identification of the Expert Panel

The members who participate in the voting process of the Delphi survey are called panelists. The panel will consist of clinical or research-based experts with relevant backgrounds in primary care. A homogeneous panel of medical experts in primary care will be selected according to predefined criteria to achieve generalization of consensus and assessment of the relevance and feasibility of management recommendations.

### Recruitment

Participants will be identified through self-nomination and identification by the project team based on their track record. Individuals identified will be sent a personalized invitation letter. Self-nomination as Delphi panelists will occur through networks for primary care physicians. The institute has established a network of primary care physicians who are engaged in teaching and research activities. A unique identifier will be assigned to each participant to enable monitoring of completion and ensure anonymity.

### Eligibility Screen

All identified experts will be invited to participate. The potential participants will receive materials to inform them about the study. They will be sent an invitation letter, a participant information sheet, and a participant consent form. Panelists will be provided specific information about the project and asked for their consent to participate. Experts will be screened at this stage for eligibility to participate, which includes holding a Foederatio Medicorum Helveticorum specialization or title in primary care medicine and having an intrinsic interest in this user population and in anabolic agents. Potential participants will be asked to declare any possible conflicts of interest. Participants will be asked to commit to completing all required rounds of the Delphi process; incentives will be provided only upon completion of all Delphi rounds. Participants will be able to withdraw from the project at any time. Potential participants will be provided with a clear explanation of the anticipated process, including the requirement that participation will be over a period of months with multiple rounds of questioning and feedback. They will be asked to sign a written consent form to be involved in the Delphi process.

### Panel Size

There is no standard sample size for the panel members. However, due to data management challenges and logistic issues, a panel size of approximately 30 to 50 members is recommended as optimal for conducting homogeneous Delphi rounds according to the literature, with a minimum number of 12 members [47,52]. According to a systematic review that analyzed quality indicators in Delphi studies, most panels consisted of 11 to 31 members, with a median of 17 members [53]. This study will aim to recruit a minimum of 25 participants.

### Design and Content of the Survey

The study will be conducted entirely on the web. The Delphi survey rounds will be designed and administered electronically using an established and validated Delphi Manager software. The previously determined questionnaire items will be incorporated into the initial questionnaire. Quantitative Delphi rounds will then be conducted among the expert panelists. These rounds will ask experts to rate items in terms of relevance (appropriateness in primary care) and feasibility (implementability in primary care) based on predetermined concepts. Quantitative rounds will also provide space for additional (qualitative) comments, allowing experts to suggest amendments or additions to the items. Qualitative feedback will be mapped by disease area and discussed among the research steering group based on relevance and feasibility for inclusion in subsequent Delphi rounds.

Each round is estimated to take up to 60 minutes to complete and will be accessible for 3 weeks. To enhance the response rate, Delphi panelists will receive weekly reminders if the survey is not completed. Results will be analyzed for at least 2 weeks between rounds. Subsequent rounds will provide detailed feedback about responses to previous rounds, including individual and group responses or consensus and median (IQR) for each item. Participants will receive up to 3 email reminders to complete each round.

### Rating Process

Participants will be asked to rate each item for relevance and feasibility in 3 rounds of the Delphi survey using a 5-point Likert scale (ie, relevance: 1=irrelevant and 5=relevant; feasibility: 1=not feasible and 5=feasible). Textboxes will be available for participants to provide comments, including recommendations for additional items, for each subgroup of indicators. In round 1, participants will be asked to provide demographic information once (ie, name, age, gender, professional details or area of practice, number of years of primary care practice, and level of experience with AAS users). Then, they will be asked to rate the questionnaire items. Items that reach consensus will be removed. In round 2, participants will be presented with items that did not reach consensus, as well as new items or item changes based on qualitative feedback. Furthermore, they will receive a summary feedback of previous responses from round 1 and be given the opportunity to re-evaluate or change their previous responses. In the final round, that is, round 3, the rating process from round 2 will be repeated.

### Data Analysis

Descriptive statistics will be used to summarize and analyze the data. Demographics of the Delphi panelists will be analyzed using Microsoft Excel. Aggregate results of the panelists’ responses will be analyzed for response rates, levels of relevance and feasibility for each item, and medians and IQRs [54]. The final score for relevance and feasibility will be calculated as the percentage of Delphi panelists who assigned a combined rating of 4 or 5, divided by the total number of responses, and multiplied by 100. Regarding qualitative feedback, the research team will analyze the data and discuss possible changes in items and the inclusion of new items. To maintain rigor, the response rate of each Delphi round should not fall below 70% [55]. No subsequent rounds will be launched if the response rate of the previous round is below that threshold. In case of missing data, those responses will be removed, and datasets for each item will be analyzed with a reduced sample size if the response rate is 70% or more.

### Closing Criteria

The levels of consensus in the Delphi methodology can vary depending on the size of the expert panel and the aim of the research. Each item will require 75% or greater agreement in both relevance and feasibility to achieve consensus [56]. The final consensus will present issues upon which the experts agree (consensus), along with the items that remain controversial (nonconsensus).

### Data Management

Demographic data collected from Delphi panelists and consent forms will be stored in a Microsoft Word or PDF document. An Excel spreadsheet will be created in which participant names will be assigned an ID number. Survey files will be assigned using the participant’s number to ensure anonymity. Hard copies of documents will be stored in a locked filing cabinet at the University of Zurich. Statistical analyses will be performed using R statistical software (version 4.2.2; R Foundation for Statistical Computing) [57]. Digital data will be stored on a secure cloud-based system at the University of Zurich.

### External Validation

The final draft of the resulting best practice guidance will be reviewed by an external board before publication and dissemination. Approval will be sought from the quality commission of the Swiss Society of General Internal Medicine, the Swiss Society of Addiction Medicine, and the Sport and Exercise Medicine Switzerland (SEMS).

### Ethical Considerations

This Delphi study was reviewed by the Cantonal Ethics Committee Zurich, Switzerland (Business Administration System for Ethics Committees, BASEC-Nr Req-2024-00643) and was determined not to fall within the scope of the Human Research Act.

Before the Delphi rounds, panelists will receive an email providing detailed information regarding the investigators, the purpose of the study, a description of the research, participant involvement, risks and potential benefits, confidentiality, authorization and withdrawal procedures, compensation, the voluntary nature of participation, assurance of anonymity, a request for consent, and an approximate estimation of the survey’s duration.

During the Delphi rounds, panelists will remain anonymous to each other but not to the researchers. Data will be anonymized and will be accessible only to authorized study personnel. Anonymity of the panelists will be guaranteed throughout the Delphi rounds. Upon conclusion, panelists who complete all Delphi rounds will be offered the choice to remain anonymous or receive acknowledgment in the publication for their participation (ie, name and organization). All medical information obtained within this study will be considered confidential.

Small incentives will be provided for expert panelists for their contributions in the Delphi rounds. A total financial incentive of CHF 500 (US $628.78) will be given to each panel expert upon the completion of all Delphi rounds at the end of the study.

Legal aspects and professional ethics principles in medicine need to be considered when providing medical care to individuals engaged in recreational sports. Regarding the implementation of clinical practice in Switzerland, we have previously discussed the legal aspects of providing medical care elsewhere and demonstrated its feasibility within the legal scope at the Arud Centre [11,29]. Importantly, the medical support and care provided are not intended to support doping but rather to combat addiction and problematic consumption based on a clear medical indication. Taking legal and professional principles into account, it is essential for health care professionals to be aware of the legal limitations of providing care to protect themselves from possible legal repercussions.

## Results

As of February 2025, the research has commenced with the scoping literature review (ie, systematic identification of the problem area), and no Delphi participants have been recruited. The tentative timeline is outlined in Textbox 1. Results are expected to be published in the summer of 2026. The SEMS granted funding for the conduct of this research in September 2024. Ethics approval for the conduct of this study was received in May 2024.

**Delphi study timeline.**

**Study protocol development (before 2025)**

- Identification of problem area: January 2025 to July 2025
- Survey development and selection of expert panel: August 2025 to December 2025
- Iterative quantitative Delphi rounds: January 2026 to April 2026
- Manuscript development: May 2026 to July 2026

## Discussion

### Anticipated Findings

This study protocol describes the design for a Delphi study aimed at reaching consensus among health care experts in primary care on a set of relevant and feasible measures to optimize the provision of current medical care for AAS users in a recreational sports setting. Findings from this Delphi consensus study will address knowledge gaps in the medical literature and provide high-quality recommendations for future use by health care providers in primary care. Measures to be evaluated in this Delphi study will be derived from a mixed methods approach, incorporating a review of existing medical literature. This study will further build on these initial results and close existing knowledge gaps with opinions from medical experts in specialized medical fields, peers, and anabolic steroid management specialists with clinical experience in this population. This mixed methods approach is critical for addressing the many medical challenges arising from this substance use.

Principal findings from reviewing the medical literature demonstrate that the consumption of AASs is associated with many harms, including cardiovascular complications and the development of cardiomyopathy [17,33,42,58-60]; liver and kidney injury [16,61-64]; endocrine, reproductive, and metabolic disorders [15,40,46,65-67]; infectious diseases [68,69]; negative mental health outcomes [70-72]; and even cancer development [14,16,73]. Furthermore, health risks may arise and culminate in this population due to consumption practices (ie, supraphysiologic doses of AASs in complex user patterns, length of consumption over weeks or months, use of extensive polypharmacy, and concomitant illicit substance use [74-79]); high-risk behavioral practices (eg, injection-related, sexual, exercise, and nutritional practices [62,80]); addiction-related medical factors (eg, anabolic steroid dependence, muscle dysmorphia, and concomitant substance use disorders [65,76,81]); or individual factors (eg, genetic, sociodemographic, and economic factors [19]). The great extent of harm to multiple organ systems and further risks associated with AAS consumption emphasize the need for a multidisciplinary approach in developing comprehensive medical guidance, which represents a strength of this study design. The primary care setting appears to be most suitable for providing comprehensive medical care to people using these substances, as it offers low-threshold access to health care, provides long-term and chronic care, and coordinates within the health care sector when dealing with multiple complications from this substance use [29].

This study design offers additional strengths for the development of clinical guidance. The Delphi technique is an appropriate tool to develop consensus among diverse experts because it offers anonymity to participants and minimizes bias from possibly dominant experts compared to other consensus development methods (ie, focus groups). The use of scoring criteria for approval of each consensus item is expected to ensure its relevance and feasibility in clinical practice. Furthermore, participants can review individual and group results from previous rounds and reassess their responses. In addition to seeking consensus from health care experts, the study team will seek broad institutional approval from different medical associations (ie, Swiss Society of General Internal Medicine, Swiss Society of Addiction Medicine, as well as SEMS). This is a unique attribute of this Delphi study design regarding future implementation and rollout of the final guidance. Results will be disseminated in peer-reviewed scientific journals; presented at national and international conferences; and circulated through professional organizations relevant to primary care medicine, sports medicine, and addiction medicine. The final manuscript will also be circulated to all participants. Another important feature of this project is to create awareness of the legal aspects that need to be considered when providing medical care to AAS users and the urgent need for policy changes. The development of adequate best practice medical guidance can further strengthen the alliance between different stakeholders and medical experts for future guidance on policy changes by demonstrating the extent of the problems that arise through this substance use and its possible negative public health outcomes.

Limitations of the Delphi technique need to be mentioned. The study methodology does not involve direct interactions with the panelists, which may limit their ability to generate ideas during the consensus process. Furthermore, this study is designed specifically for the Swiss primary care context and may not represent the primary care setting in other countries, thus challenging the generalization of results.

### Conclusions

The use of AASs among younger male individuals in recreational sports has become a major global health concern. The nonmedical use of these substances may be associated with a sequela of harms on many organ systems, as well as substance dependence and many more risks that can arise from a wide range of high-risk behaviors. The optimal provision of medical care for this population is challenged by limited data in the medical literature and a lack of consensus among health care experts. The Delphi consensus study, comprising a mixed methods study design, is an appropriate tool to gain consensus from different stakeholders and health care experts. Results from this study will create a consensus-based best current clinical guidance for primary care providers in Switzerland. The primary care setting may be the best place to provide integrated and comprehensive medical care and harm-reducing measures to patients with AAS-related health complications.

## Appendix

Multimedia Appendix 1 PRISMA-ScR checklist.

Multimedia Appendix 2 Scoping literature review search strategy.

## Abbreviations

AAS: anabolic androgenic steroid
IPED: image- and performance-enhancing drug
PRISMA-ScR: Preferred Reporting Items for Systematic Reviews and Meta-Analyses extension for Scoping Reviews
SEMS: Sport and Exercise Medicine Switzerland

## Data Availability

No underlying data are available for this paper because no datasets were generated or analyzed during this study.
