# Supplementary material for: Best Practice Guidance for Male Individuals Using Anabolic Androgenic Steroids in Recreational Sports Within Primary Care: Protocol for a Modified Delphi Consensus Study
Source: JMIR Res Protoc. 2025 Aug 18;14:e65233. doi: 10.2196/65233 (PMC12402736; doi:10.2196/65233)
Supplement: Multimedia Appendix 2 [file resprot_v14i1e65233_app2.docx]

# Supplementary File 1: Literature search strategy

## Literature search 1 - Search strategy for screening for anabolic steroid use

PubMed Search: **((anabolic steroids) AND (screening)) OR (identification)**Filters: **from 2005 - 2025**

((("anabolic androgenic steroids"[MeSH Terms] OR ("anabolic"[All Fields] AND "androgenic"[All Fields] AND "steroids"[All Fields]) OR "anabolic androgenic steroids"[All Fields] OR ("anabolic"[All Fields] AND "steroids"[All Fields]) OR "anabolic steroids"[All Fields]) AND ("diagnosis"[MeSH Subheading] OR "diagnosis"[All Fields] OR "screening"[All Fields] OR "mass screening"[MeSH Terms] OR ("mass"[All Fields] AND "screening"[All Fields]) OR "mass screening"[All Fields] OR "early detection of cancer"[MeSH Terms] OR ("early"[All Fields] AND "detection"[All Fields] AND "cancer"[All Fields]) OR "early detection of cancer"[All Fields] OR "screen"[All Fields] OR "screenings"[All Fields] OR "screened"[All Fields] OR "screens"[All Fields])) OR ("identifed"[All Fields] OR "identification, psychological"[MeSH Terms] OR ("identification"[All Fields] AND "psychological"[All Fields]) OR "psychological identification"[All Fields] OR "identification"[All Fields] OR "identifications"[All Fields])) AND (2004:2024[pdat])

**Translations**

**anabolic steroids:** "anabolic androgenic steroids"[MeSH Terms] OR ("anabolic"[All Fields] AND "androgenic"[All Fields] AND "steroids"[All Fields]) OR "anabolic androgenic steroids"[All Fields] OR ("anabolic"[All Fields] AND "steroids"[All Fields]) OR "anabolic steroids"[All Fields]

**screening:** "diagnosis"[Subheading] OR "diagnosis"[All Fields] OR "screening"[All Fields] OR "mass screening"[MeSH Terms] OR ("mass"[All Fields] AND "screening"[All Fields]) OR "mass screening"[All Fields] OR "early detection of cancer"[MeSH Terms] OR ("early"[All Fields] AND "detection"[All Fields] AND "cancer"[All Fields]) OR "early detection of cancer"[All Fields] OR "screen"[All Fields] OR "screenings"[All Fields] OR "screened"[All Fields] OR "screens"[All Fields]

**identification:** "identifed"[All Fields] OR "identification, psychological"[MeSH Terms] OR ("identification"[All Fields] AND "psychological"[All Fields]) OR "psychological identification"[All Fields] OR "identification"[All Fields] OR "identifications"[All Fields]

###

## Literature search 2 - Side effects and complications from use of anabolic androgenic steroids

PubMed Search: **((anabolic steroids) AND (side effects)) OR (complications)** Filters: **from 2005 - 2025**

((("anabolic androgenic steroids"[MeSH Terms] OR ("anabolic"[All Fields] AND "androgenic"[All Fields] AND "steroids"[All Fields]) OR "anabolic androgenic steroids"[All Fields] OR ("anabolic"[All Fields] AND "steroids"[All Fields]) OR "anabolic steroids"[All Fields]) AND ("adverse effects"[MeSH Subheading] OR ("adverse"[All Fields] AND "effects"[All Fields]) OR "adverse effects"[All Fields] OR ("side"[All Fields] AND "effects"[All Fields]) OR "side effects"[All Fields])) OR ("complicances"[All Fields] OR "complicate"[All Fields] OR "complicated"[All Fields] OR "complicates"[All Fields] OR "complicating"[All Fields] OR "complication"[All Fields] OR "complication s"[All Fields] OR "complications"[MeSH Subheading] OR "complications"[All Fields])) AND (2004:2024[pdat])

**Translations**

**anabolic steroids:** "anabolic androgenic steroids"[MeSH Terms] OR ("anabolic"[All Fields] AND "androgenic"[All Fields] AND "steroids"[All Fields]) OR "anabolic androgenic steroids"[All Fields] OR ("anabolic"[All Fields] AND "steroids"[All Fields]) OR "anabolic steroids"[All Fields]

**side effects:** "adverse effects"[Subheading] OR ("adverse"[All Fields] AND "effects"[All Fields]) OR "adverse effects"[All Fields] OR ("side"[All Fields] AND "effects"[All Fields]) OR "side effects"[All Fields]

**complications:** "complicances"[All Fields] OR "complicate"[All Fields] OR "complicated"[All Fields] OR "complicates"[All Fields] OR "complicating"[All Fields] OR "complication"[All Fields] OR "complication's"[All Fields] OR "complications"[Subheading] OR "complications"[All Fields]

## Literature search 3 - Management recommendation of side effects and complications from use of anabolic androgenic steroids

PubMed Search: **(anabolic steroids) AND (management)** Filters: **from 2005 - 2025**

(("anabolic androgenic steroids"[MeSH Terms] OR ("anabolic"[All Fields] AND "androgenic"[All Fields] AND "steroids"[All Fields]) OR "anabolic androgenic steroids"[All Fields] OR ("anabolic"[All Fields] AND "steroids"[All Fields]) OR "anabolic steroids"[All Fields]) AND ("manage"[All Fields] OR "managed"[All Fields] OR "management s"[All Fields] OR "managements"[All Fields] OR "manager"[All Fields] OR "manager s"[All Fields] OR "managers"[All Fields] OR "manages"[All Fields] OR "managing"[All Fields] OR "managment"[All Fields] OR "organization and administration"[MeSH Terms] OR ("organization"[All Fields] AND "administration"[All Fields]) OR "organization and administration"[All Fields] OR "management"[All Fields] OR "disease management"[MeSH Terms] OR ("disease"[All Fields] AND "management"[All Fields]) OR "disease management"[All Fields])) AND (2004:2024[pdat])

**Translations**

**anabolic steroids:** "anabolic androgenic steroids"[MeSH Terms] OR ("anabolic"[All Fields] AND "androgenic"[All Fields] AND "steroids"[All Fields]) OR "anabolic androgenic steroids"[All Fields] OR ("anabolic"[All Fields] AND "steroids"[All Fields]) OR "anabolic steroids"[All Fields]

**management:** "manage"[All Fields] OR "managed"[All Fields] OR "management's"[All Fields] OR "managements"[All Fields] OR "manager"[All Fields] OR "manager's"[All Fields] OR "managers"[All Fields] OR "manages"[All Fields] OR "managing"[All Fields] OR "managment"[All Fields] OR "organization and administration"[MeSH Terms] OR ("organization"[All Fields] AND "administration"[All Fields]) OR "organization and administration"[All Fields] OR "management"[All Fields] OR "disease management"[MeSH Terms] OR ("disease"[All Fields] AND "management"[All Fields]) OR "disease management"[All Fields]
